# Hypofibrinolytic State in Subjects with Type 2 Diabetes Mellitus Aggravated by the Metabolic Syndrome before Clinical Manifestations of Atherothrombotic Disease

**DOI:** 10.1155/2017/6519704

**Published:** 2017-02-08

**Authors:** Elsa Aburto-Mejía, David Santiago-Germán, Manuel Martínez-Marino, María Eugenia Galván-Plata, Eduardo Almeida-Gutiérrez, Mardia López-Alarcón, Jesús Hernández-Juárez, Antonio Alvarado-Moreno, Alfredo Leaños-Miranda, Abraham Majluf-Cruz, Irma Isordia-Salas

**Affiliations:** ^1^Servicio de Medicina Interna, UMAE Hospital de Especialidades, CMN Siglo XXI, Instituto Mexicano del Seguro Social, Ciudad de México, Mexico; ^2^Servicio de Urgencias, H.G.R. No. 1 “Dr. Carlos Mac Gregor Sánchez Navarro”, Instituto Mexicano del Seguro Social, Ciudad de México, Mexico; ^3^Servicio de Neurología, UMAE Hospital de Especialidades, Instituto Mexicano del Seguro Social, Ciudad de México, Mexico; ^4^Coordinación de Investigación en Salud, Instituto Mexicano del Seguro Social, Ciudad de México, Mexico; ^5^Unidad de Investigación en Nutrición Médica, CMN Siglo XXI, Instituto Mexicano del Seguro Social, Ciudad de México, Mexico; ^6^Unidad de Investigación Médica en Trombosis, Hemostasia y Aterogénesis, H.G.R. No. 1 “Dr. Carlos Mac Gregor Sánchez Navarro”, Instituto Mexicano del Seguro Social, Ciudad de México, Mexico; ^7^Unidad de Investigación Médica en Medicina Reproductiva, UMAE H.G.O. No. 4, Instituto Mexicano del Seguro Social, Ciudad de México, Mexico

## Abstract

*Background*. Metabolic and genetic factors induce plasminogen activator inhibitor type-1 (PAI-1) overexpression; higher PAI-1 levels decrease fibrinolysis and promote atherothrombosis.* Aim*. To assess PAI-1 antigen levels among subjects with type 2 diabetes mellitus (T2DM) plus Metabolic Syndrome (MetS) before clinical manifestations of atherothrombosis and the contribution of metabolic factors and 4G/5G polymorphism of PAI-1 gene on the variability of PAI-1.* Methods*. We conducted an observational, cross-sectional assay in a hospital in Mexico City from May 2010 to September 2011. MetS was defined by the International Diabetes Federation criteria. PAI-1 levels and 4G/5G polymorphism were determined by ELISA and PCR-RFLP analysis.* Results*. We enrolled 215 subjects with T2DM plus MetS and 307 controls. Subjects with T2DM plus MetS had higher PAI-1 levels than the reference group (58.4 ± 21 versus 49.9 ± 16 ng/mL, *p* = 0.026). A model with components of MetS explained only 12% of variability on PAI-1 levels (*R*^2^ = 0.12; *p* = 0.001), with *β* = 0.18 (*p* = 0.03) for hypertension, *β* = −0.16 (*p* = 0.05) for NL HDL-c, and *β* = 0.15 (*p* = 0.05) for NL triglycerides.* Conclusion*. Subjects with T2DM plus MetS have elevated PAI-1 levels before clinical manifestations of atherothrombotic disease. Metabolic factors have a more important contribution than 4G/5G polymorphism on PAI-1 plasma variability.

## 1. Introduction

Plasminogen activator inhibitor type-1 (PAI-1) is the principal inhibitor of fibrinolysis [1]. PAI-1 concentrations are determined by age, gender, ethnicity, circadian rhythm, adipose tissue distribution, sympathetic nerve activity, smoking status, and chronic inflammation [1]. Higher levels of PAI-1 are associated with an increased risk of atherothrombosis by at least two mechanisms: by decreased fibrinolysis and by inhibition of vascular smooth muscle cell migration and proliferation, predisposing to formation of atheroma plaques prone to rupture, by a thin fibrous cap of collagen separating the lipid core from the arterial lumen [2, 3]. Insulin resistance, obesity, dyslipidemia, and endothelial dysfunction also induce PAI-1 overexpression and are associated with an augmented cardiovascular risk [4]. The cluster of those traits in a same individual is known as the Metabolic Syndrome [5, 6]. The increase in the number of metabolic risk factors in the same person increases the risk for ischemic heart disease and stroke [5, 6]. In Mexico, more than 17 million adults have the Metabolic Syndrome, and 3.5 million are already diagnosed with diabetes [7]. Previously, we reported a frequency of 68% of the Metabolic Syndrome in an urban Mexican sample [8]. Furthermore, high prevalence of patients with coronary artery disease in our country has Metabolic Syndrome (43.4%) [9].

Paradoxically, the reduction of blood glucose levels does not decrease cardiovascular event incidence in patients with type 2 diabetes mellitus (T2DM), suggesting that impaired synthetic and secretory capacity of endothelial cells, increased platelet reactivity, cellular stress, and increased circulating procoagulant and proinflammatory molecules contribute to the enhanced cardiovascular risk in diabetic subjects [10]. In addition, ~50% of individuals with atherothrombotic disease are lacked of traditional cardiovascular risk factors, suggesting a genetic contribution [11]. Previously, we identified the allele 4G of the PAI-1 gene as an independent risk factor for ST elevation myocardial infarction in young Mexican individuals and higher PAI-1 plasma concentrations in homozygous for the allele 4G [12]. In contrast, the allele 4G was not associated with an increased risk of ischemic stroke in a young Mexican sample [13]. PAI-1 plasma concentrations vary across populations, at least in part by differences in ethnicity and genetic background [14].

Therefore, the purpose of the present research was to assess the PAI-1 plasma concentrations among subjects with T2DM aggravated by the Metabolic Syndrome before clinical manifestations of atherothrombotic disease in addition to evaluating the contribution of metabolic factors and the 4G/5G polymorphism of the PAI-1 gene on the plasma variability of PAI-1 among a sample of Mexican subjects with T2DM plus the Metabolic Syndrome.

## 2. Materials and Methods

We conducted an observational, cross-sectional assay in a secondary care level hospital at Mexico City from May 2010 to September 2011. We screened consecutive apparently healthy members of the medical staff, relatives of outpatients whom came to medical consultation, and those who were in routine follow-up for diabetes mellitus. The recruitment was made by invitation through printed announcements and personal appeal to people to participate in the survey if they were interested to know their glucose tolerance status and cardiovascular risk factors. We included all individuals ≥20 years old who accepted to participate. Informed written consent was obtained from all subjects before enrollment. The study protocol was reviewed and approved by the Human Ethical Committee and Medical Research Council of the Mexican Institute of Social Security (IMSS) and conforms to the ethical guidelines of the 1975 Declaration of Helsinki.

The exclusion criteria were subjects with previous or current diagnosis of atherothrombotic disease (i.e., myocardial infarction, angina, stroke, transient ischemic attack, and peripheral artery disease), cancer, autoimmune disorders, acute and chronic infectious diseases, and hepatic or renal failure, those under immunosuppressive therapy, and transplant receivers.

Demographic and clinical data were collected using a questionnaire in a private interview performed by a physician; the information contained in the survey included age, gender, smoking status, previous diseases, and familial history of diabetes and cardiovascular disease. Anthropometric parameters were taken from all the participants interviewed; the same physician measured all the subjects. Waist circumference (WC) was measured at the midpoint between the last rib and the iliac crest with subjects standing and wearing only undergarments. Body weight was measured by precision scale, while subjects were minimally clothed without shoes. Height was measured in a standing position without shoes using tape meter, while the shoulders were in a normal state. Body mass index (BMI) was calculated as weight in kilograms divided by height in meters squared. Blood pressure was measured after a 5-minute rest in a seated position. Two readings were taken in 5-minute interval between these two separated measurements, and thereafter the mean of the two measurements was considered to be the participant's blood pressure. Blood samples were obtained from all the participants by puncture of the antecubital vein at morning (between 8:00 and 10:00 am) after an overnight fast of at least 8 hours. Biochemical measurements included fasting plasma glucose (FPG), glycosylated hemoglobin (HbA1c), high-density lipoprotein cholesterol (HDL-c), and triglycerides. Buffy coat and plasma were collected and frozen at −70°C for subsequent biochemical and genetic analysis. PAI-1 serum levels and 4G/5G polymorphism of PAI-1 gene were determined in all subjects with T2DM plus the Metabolic Syndrome in accord with the International Diabetes Federation (IDF) criteria and the reference group.

T2DM was defined as a FPG ≥ 126 mg/dL or HbA1c ≥ 6.5% or previous diagnosis [15]. The Metabolic Syndrome was defined as central obesity (a waist circumference ≥90 cm for men and ≥80 cm for women according to IDF criteria for Hispanic population) plus any one of the following factors: (1) raised triglycerides ≥ 150 mg/dL or under specific drug treatment; (2) reduced HDL-c <40 mg/dL in males and <50 mg/dL in females or under specific drug treatment; and (3) a systolic blood pressure ≥130 mm Hg or diastolic blood pressure ≥85 mm Hg or use of antihypertensive treatment [16]. The subjects were considered smokers if they were currently smoking (regularly or occasionally, including also former smokers defined as people who stopped smoking at least one year before the examination). A familial history of cardiovascular disease was defined as acute myocardial infarction, stroke, or sudden death in a first-degree male relative below 55 years of age or a female relative below 65 years of age. Subjects without T2DM and the Metabolic Syndrome were considered as the reference group.

### 2.1. Determination of PAI-1 Antigen Plasma Levels

PAI-1 plasma concentrations were determined from blood samples collected between 8:00 and 10:00 am to avoid variations due to circadian rhythm and stored in tubes containing citrate as anticoagulant. Samples were centrifuged at 3000*g* for 25 min at 4°C to avoid the contamination of plasma with platelets. Then, they were stored in aliquots of 0.5 mL at −70°C until use. The plasma concentration of PAI-1 was determined immunoenzymatically by enzyme-linked immunosorbent assay (ELISA) (Coaliza PAI-1, Chromogenix, Milan, Italy).

### 2.2. Deoxyribonucleic Acid Extraction and Genotyping

Genomic deoxyribonucleic acid (DNA) was obtained from leukocyte concentrate of peripheral blood using the commercial QIAamp DNA Blood Mini Kit (QIAGEN, Hilden, Germany) according to the manufacturer's instructions. Genotyping of the 4G/5G polymorphism in the PAI-1 promoter region was performed by polymerase chain reaction (PCR) using the following oligonucleotides: 5′-CACAGAGAGAGTCTGGCCACGT-3′ (sense) and 5′-CCAACAGAGGACTCTTGGTCT-3′ (antisense) [17]. The reaction conditions were as follows: initial denaturation at 94°C for 3 min followed by 30 cycles of denaturation at 94°C for 30 s, alignment at 60°C for 30 s, and an extension step at 72°C for 30 s, followed by a final linear extension step at 72°C for 1 min. Amplification products of 99 bp (5G) and 98 bp (4G) were obtained. The PCR products were subjected to digestion with the specific restriction enzyme* BslI* (New England Biolabs, Beverly, Massachusetts, USA) at 55°C. The DNA fragments were separated by electrophoresis in 3% agarose gel (Bio-Rad Laboratories, Hercules, California, USA) and visualized using SYBR Safe DNA Gel Stain (Invitrogen). All samples were processed in duplicate. Some samples were subject to sequencing.

### 2.3. Statistical Analysis

Continuous variables with normal distribution were expressed as mean ± standard deviation; those with nonparametric distribution were expressed as median and interquartile range. Categorical data were expressed as total number and percentage. Continuous data were submitted to normality tests; variables not normally distributed were transformed to natural logarithm (NL) before any statistical analysis. Continuous variables were compared between subjects with T2DM plus the Metabolic Syndrome and the reference group by Student's *t*-test. Categorical variables were compared between both groups by *X*^2^ test. The allele frequency of the 4G/5G polymorphism conforming to Hardy-Weinberg equilibrium proportions was tested using *X*^2^ test. PAI-1 antigen plasma levels were compared between homozygous 4G/4G, 5G/5G, and heterozygous 4G/5G carriers in diabetic subjects plus the Metabolic Syndrome and the reference group using one-way analysis of variance (ANOVA). A Pearson correlation analysis was performed to evaluate the association between continuous explanatory variables (such as age, WC, HDL-c, triglycerides, and FPG) and PAI-1 plasma levels. A Spearman correlation analysis was performed to evaluate the relationship between categorical explanatory variables (such as gender, elevated blood pressure, smoking status, and genotype) and PAI-1 plasma levels. A multivariable linear regression analysis was performed to develop a model that includes only the variables with linear correlation in the bivariate analysis (as the explanatory variables) with PAI-1 plasma levels (as the response variable) to estimate the independent contribution of each feature to variation in PAI-1 plasma concentrations. A *p* value ≤ 0.05 (two-tailed) was considered statistically significant. All statistical analyses were performed using SPSS (Statistical Package for the Social Sciences) statistical software package (version 15: SPSS Inc., Chicago, IL, USA).

## 3. Results

**Figure 1:**
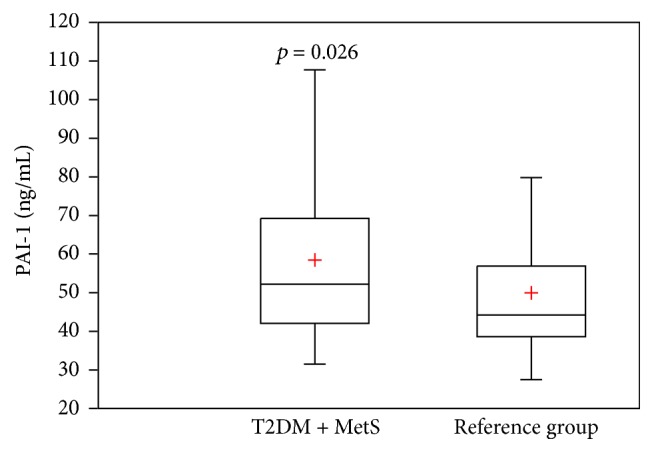
Box plots showing PAI-1 antigen plasma levels between diabetic subjects plus the Metabolic Syndrome and the reference group. + = Mean; *p* = *p* value; PAI-1 = plasminogen activator inhibitor type-1; T2DM = type 2 diabetes mellitus; MetS = the Metabolic Syndrome.

A total sample of 215 subjects with T2DM plus the Metabolic Syndrome was recruited among an urban population from Mexico City between May 2010 and September 2011. We enrolled 307 subjects without T2DM or the Metabolic Syndrome as the reference group. Comparison of clinical and biochemical characteristics between the two groups is shown in Table 1. Individuals with T2DM plus the Metabolic Syndrome were older (with a mean age of 56 ± 10 versus 48.5 ± 13.7 years, *p* < 0.0001), with higher levels of FPG (136 (112–181) versus 90 (85–95) mg/dL, *p* < 0.0001), HbA1c (5.4 (4.4–7.5) versus 3.9 (3.6–4.2) %, *p* < 0.0001), BMI (30.8 ± 5 versus 26.5 ± 4.2 kg/m^2^, *p* < 0.0001), WC (100 ± 9.9 versus 87.4 ± 11.9 cm, *p* < 0.0001), and triglycerides (205 (134–270) versus 118 (88–150) mg/dL, *p* < 0.0001) and lower levels of HDL-c (35 (30–41) versus 46.4 (37.2–55.4) mg/dL, *p* < 0.0001), when compared with the reference group as was expected. The group of subjects with T2DM plus the Metabolic Syndrome had central obesity plus at least one of the following conditions: 87.9% (*n* = 189) had lowered HDL-c, 70% (*n* = 152) had elevated triglycerides, and 60% (*n* = 129) had elevated blood pressure. There were no differences regardless of gender, smoking status, and familial history of atherothrombotic disease between both groups. There was a statistical significant difference in PAI-1 plasma levels between subjects with T2DM plus the Metabolic Syndrome and the reference group (58.4 ± 21 versus 49.9 ± 16 ng/mL, *p* = 0.026) Figure 1.

**Figure 2:**
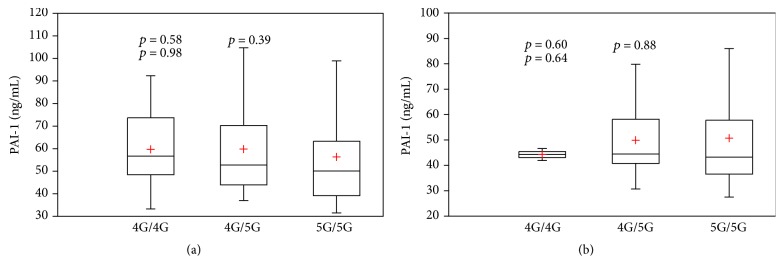
Box plots showing PAI-1 antigen plasma levels between diabetic subjects plus the Metabolic Syndrome (a) and the reference group (b), according to genotype distribution of -675 4G/5G polymorphism of the PAI-1 gene. (a) = 4G/4G versus 5G/5G, *p* = 0.58; 4G/4G versus 4G/5G, *p* = 0.98; 4G/5G versus 5G/5G, *p* = 0.39, (b) = 4G/4G versus 5G/5G, *p* = 0.60; 4G/4G versus 4G/5G, *p* = 0.64; 4G/5G versus 5G/5G, *p* = 0.88; + = mean; *p* = *p* value; PAI-1 = plasminogen activator inhibitor type-1.

The genotype distribution of 4G/5G polymorphism of PAI-1 gene in the group with T2DM plus the Metabolic Syndrome was 4G/4G, 11.6% (*n* = 25), 4G/5G, 48.4% (*n* = 104), and 5G/5G, 40% (*n* = 86), with an allelic frequency of 35.8% (*n* = 154) for the allele 4G (Table 2). There were no statistical differences in genotype distribution and allelic frequency when compared with the reference group. Subjects with T2DM plus the Metabolic Syndrome homozygous for the allele 4G/4G had the highest PAI-1 plasma levels (56.7 (48.5–73.7) ng/mL), followed by the heterozygous allele 4G/5G (52.7 (43.9–70.2) ng/mL), with the lowest concentration for the homozygous allele 5G/5G (50.1 (39.1–63.3) ng/mL), but without significant statistical difference between the three genotypes by ANOVA test (4G/4G versus 5G/5G, *p* = 0.58; 4G/5G versus 5G/5G, *p* = 0.39; 4G/4G versus 4G/5G, *p* = 0.98) (Figure 2(a)). The reference group showed similar PAI-1 plasma levels for the three genotypes; the homozygous 4G/4G had a median of 44.2 (43–45.4) ng/mL, the heterozygous 4G/5G had a median of 44.4 (40.7–58.1) ng/mL, and the 5G/5G had a median of 43.2 (36.5–57.7) ng/mL, without statistical significance (4G/4G versus 5G/5G, *p* = 0.60; 4G/5G versus 5G/5G, *p* = 0.88; 4G/4G versus 4G/5G, *p* = 0.64) (Figure 2(b)).

Correlation coefficients between PAI-1 plasma levels and metabolic factors in subjects with T2DM plus the Metabolic Syndrome are shown in Table 3. PAI-1 plasma concentrations were positively associated with elevated natural logarithm of triglycerides (*r* = 0.24; *p* = 0.004) and hypertension (*r*_*s*_ = 0.22; *p* = 0.01). In contrast, there was a negative relationship between PAI-1 plasma levels and natural logarithm of HDL-c (*r* = −0.21; *p* = 0.01). The rest of the variables did not have linear correlation: abdominal obesity measured by WC (*r* = 0.15; *p* = 0.08), FPG (*r* = 0.01; *p* = 0.88), age (*r* = −0.05; *p* = 0.53), gender (*r* = 0.27; *p* = 0.53), smoking status (*r* = 0.07; *p* = 0.42), and 4G/5G polymorphism (*r* = 0.06; *p* = 0.45).

The variability on PAI-1 plasma levels explained by the components of the Metabolic Syndrome is shown in Table 4. The model included NL HDL-c, NL triglycerides, and hypertension; this model only explained 12% of variance in PAI-1 plasma levels (*R*^2^ = 0.12; *p* = 0.001). Components of the Metabolic Syndrome were much more determinant in PAI-1 variability than the 4G/5G polymorphism, with a standardized correlation coefficient (*β*) statistically meaningful for hypertension: *β* = 0.18 (0.35 to 0.012), *p* = 0.03*; β* = −0.16 (*−*0.33 to* −*0.01 for NL HDL-c), *p* = 0.05; and *β* = 0.15 (0.01 to 0.33), *p* = 0.05, for NL triglycerides.

## 4. Discussion

We analyzed 215 subjects with T2DM plus the Metabolic Syndrome according to IDF criteria without clinical manifestation of atherothrombotic disease from a secondary care level hospital at Mexico City and compared them with the reference group (*n* = 307). The overall sample of subjects with T2DM plus the Metabolic Syndrome had elevated waist circumference according to parameters for Hispanic population plus any one of the following factors: lowered HDL-c (87.9%), hypertriglyceridemia (70%), and hypertension (60%). Similar results were reported from a nationally representative subsample randomly selected in the Mexican National Health and Nutrition Survey 2006 (ENSANUT 2006), with prevalence of 49.8% (95% CI: 47.5 to 52.1) cases with the Metabolic Syndrome using the IDF definition regardless of geographical region and socioeconomic status, predominantly women (52.7%) [7]. The frequency of metabolic factors reported by the ENSANUT 2006 study in a subsample of subjects with T2DM plus the Metabolic Syndrome was as follows: in first place, 83% (95% CI: 76.5 to 88) for reduced HDL-c, followed by 65% (95% CI: 56.5 to 72.6) for hypertension, and 46.7% (95% CI: 38.8 to 54.7) for elevated triglycerides [7].

In the present study, the group of subjects with T2DM plus the Metabolic Syndrome showed higher mean PAI-1 antigen levels of 58.4 ± 21 ng/mL compared with the reference group (49.9 ± 16 ng/mL) with statistical significance (*p* = 0.026). Patients with T2DM plus the Metabolic Syndrome had PAI-1 antigen levels above the cutoff considered as normal (reference value: 2 to 47 ng/mL) even when some of them were under pharmacologic treatment and showed FPG and HbA1c levels under ranges considered as adequate. Metabolic factors frequencies and PAI-1 plasma concentrations vary among populations. PAI-1 was dramatically higher in Italian Caucasian subjects with obesity and the Metabolic Syndrome by the National Cholesterol Education Program Third Adult Treatment Panel (NCEP ATP-III) definition when compared with healthy subjects without obesity (*p* < 0.0001) [18]. A previous report from European based-sample population showed a positively and independently association between PAI-1 antigen levels and the presence of the Metabolic Syndrome according to the NCEP ATP-III criteria, reporting a median of 126 (81.4–194.2) ng/mL for those subjects with the Metabolic Syndrome versus 57.3 (35.5–99.7) ng/mL without it (*p* < 0.001) [19]. In contrast, data from multicentric cross-sectional Spanish population-based survey reported higher levels of PAI-1 in the presence of the Metabolic Syndrome or diabetes mellitus in the bivariate analysis but without statistical significance in the multivariate analysis [20]. In a sample of Malaysian subjects, there was no difference in PAI-1 antigen levels when diabetic subjects with the Metabolic Syndrome were compared against normal individuals, with a median of 28.4 (26.5–30.5) ng/mL versus 30.2 (27.1–33.7) ng/mL, respectively [21]. The variability of PAI-1 antigen levels between populations of subjects with the Metabolic Syndrome may be related to differences in (1) selection criteria for the Metabolic Syndrome, (2) the prevalence of components of the Metabolic Syndrome in the population, (3) the sample size, and (4) the lack of analysis for the effect of pharmacologic therapy in patients with dyslipidemia, hypertension, and T2DM. The pleiotropic effects of statins in reduction of cardiovascular events beyond blood cholesterol reduction include an antithrombotic property of most statins except pravastatin which downregulate the expression of PAI-1 via inhibition of Rho family proteins [22]. Also, clinical trials suggest that Angiotensin-Converting Enzyme (ACE) inhibitors may favorably modify markers of hemostasis such as PAI-1, although the data reported by different authors are still not clear [23]. There is evidence that some molecules as Angiotensin II can act as a potent fibrogenic molecule independent of its effects on blood pressure by stimulating extracellular matrix synthesis through induction of transforming growth factor-*β* (TGF-*β*) expression and increasing PAI-1 gene transcription [24]. In contrast, there are some drugs such as pioglitazone, which not only improve insulin sensitivity but also can retard preclinical atherogenesis in patients with T2DM, at least in part by a reduction in PAI-1 expression [25]. Although in our sample some individuals were under treatment with antihypertensive drugs, hypoglycemic medication, or statins, they exhibited abnormal levels of PAI-1 when compared with the reference group, which may contribute to an increased risk for atherothrombotic disease. More studies about the pharmacologic effects in PAI-1 levels are needed.

After the inclusion of all the explanatory variables correlated with PAI-1 antigen levels in a multivariable linear regression model, we found that metabolic factors with the strongest contribution in the variability of PAI-1 antigen levels in patients with T2DM plus the Metabolic Syndrome were hypertension (*β* = 0.18; *p* = 0.03), NL HDL-c (*β* = −0.16; *p* = 0.05), and NL triglycerides (*β* = 0.15; *p* = 0.05). Elevated plasma prorenin levels are commonly found in diabetic patients; and, also, it has been demonstrated that prorenin at high concentration binds and activates prorenin/renin receptor [(p)RR] on vascular smooth muscle cells in vitro, leading to increased expression of PAI-1 via Angiotensin II-independent and dependent mechanisms, suggesting that elevated prorenin levels in diabetes may contribute to progression of atherothrombotic disease [26]. Hypertension might have a predominant role in the formation of atheroma plaque rather than rupture. In a previous report by our group, hypertension represented the second cardiovascular risk factor in patients with acute myocardial infarction [12]. In addition, treating hypertension only reduces coronary heart disease (CHD) risk by about 25%; treating hypercholesterolemia in hypertensive patients reduces CHD risk more than 35%, suggesting a relationship and a synergic effect between dyslipidemia and hypertension [27]. Both metabolic factors, hypertension and dyslipidemia, represent an important trait for the development of atherothrombosis and might contribute to an increased cardiovascular risk by mechanisms that include a hypofibrinolytic state. PAI-1 could be a novel marker for evaluation of cardiovascular risk in patients with hypertension. PAI-1 antigen levels should be monitored in hypertensive patients, and treatment should be encouraged to prevent a hypofibrinolytic state.

In subjects with T2DM and the Metabolic Syndrome, the genotype distribution was 11.6%, 48.4%, and 40% for the alleles 4G/4G, 4G/5G, and 5G/5G, respectively, with an allelic frequency of 35.8% for the risk allele 4G, with any statistical difference with the reference group. Those results are consistent with a previous report in healthy subjects from the west of Mexico (with an allelic frequency of 34.1% for the allele 4G), among a control group of young individuals (≤45 years old) in a previous publication by our group (with an allelic frequency of 28.4% for the allele 4G), and in Mexican children with obesity and without it (4G allelic frequency of 32.9% versus 26.4%) [28, 29]. In contrast, variations in prevalence of allele 4G have been reported between populations. The Insulin Resistance Atherosclerosis Study (IRAS) showed a different genotype distribution of the 4G/5G polymorphism of PAI-1 gene among African Americans (28%), Hispanics (38%), and non-Hispanic whites (52%) for the allele of risk [14]. In a sample of three different South African ethnic groups, the frequency of allele 4G was lower in the African (0.13) than Indian (0.54) or White (0.58) individuals [28]. In our sample, homozygous subjects with T2DM plus the Metabolic Syndrome with the allele 4G had the highest PAI-1 antigen levels when compared with the homozygous 5G without statistical significance (56.7 (48.5–73.7) versus 50.1 (39.1–63.3) ng/mL, *p* = 0.58). In several epidemiological, clinical, and basic studies, the allele 4G has been associated with increased PAI-1 plasma levels regardless of the effect of the Metabolic Syndrome related factors [14, 30, 31]. However, the contribution of 4G/5G polymorphism in PAI-1 variability seems to be lower. In a sample of 1328 white unrelated participants from the Framingham Heart Study, the 4G/5G polymorphism explained only 2.5% of the residual variance in circulating PAI-1 levels, with the 4G allele being associated with a higher PAI-1 concentration [32]. In a cohort of 1032 white subjects without clinical evidence of atherosclerosis from southern Italy, the contribution of 4G/5G polymorphism was small (≈1%) compared with BMI and triglycerides (20%) on PAI-1 variability [33]. A sample of 510 male survivors of myocardial infarction and 543 controls from the HIFMECH Study reported a percentage of variance explained by the 4G/5G polymorphism of 1.12% (*p* = 0.004) [34]. In our sample, the 4G/4G genotype was not correlated with PAI-1 plasma levels, and therefore it was not included in the model. Differences in the genetic background and prevalence of metabolic traits between populations are determinants in the variability of PAI-1 expression and the development of cardiovascular disease, limiting the results to a specific ethnic group.

The present study exhibits a hypofibrinolytic state in a selective group of individuals with a particular environmental and genetic background, with higher PAI-1 antigen levels before clinical manifestations of atherothrombotic disease. In a previous publication, we identified higher levels of C reactive protein and fibrinogen in T2DM individuals when compared with subjects with normal glucose tolerance. Proinflammatory conditions and prothrombotic and hypofibrinolytic state might increase the risk to develop cardiovascular disease [35].

Strengths of our research include the similarities between our sample and the population-based sample from the ENSANUT 2006 study, as well as the enrolment of subjects with similar severity of the Metabolic Syndrome. Some limitations include the lack of analysis of the effect of treatment with statins, ACE inhibitors, Angiotensin receptor blockers, and insulin sensitizing drugs in PAI-1 plasma concentration. Future analysis must include the possible effect of medication on PAI-1 variability and the relationship of PAI-1 levels in patients with hypertension.

## 5. Conclusions

Subjects with T2DM aggravated by the Metabolic Syndrome have elevated PAI-1 antigen plasma levels before clinical manifestations of atherothrombotic disease. In our sample, metabolic factors (hypertension, low HDL-c, and hypertriglyceridemia) have a more important contribution than the 4G/5G polymorphism on PAI-1 plasma levels of Mexican subjects with T2DM plus the Metabolic Syndrome. However, metabolic factors only explained 12% of PAI-1 variability.

As we have shown in previous studies, PAI-1 plasma concentrations were higher in young patients with acute myocardial infarction, and hypertension was the second more frequent cardiovascular risk factor, followed by dyslipidemia [12]. Previous and recent findings support the idea that characterization of emerging biomarkers of impaired fibrinolysis such as PAI-1 should be measured for surveillance of transition from a healthy state through the development of the Metabolic Syndrome to atherothrombotic disease and for prevention purposes in individuals with high risk of atherothrombotic disease in order to help us identify vulnerable groups for the correct targeting treatments and avoid future atherothrombotic complications such as myocardial infarction and stroke.

## Figures and Tables

**Table 1:** Comparison of clinical and biochemical characteristics between diabetic subjects with the Metabolic Syndrome and the reference group.

Characteristic	MetS	Reference group	*p* value
	*n* = 215	*n* = 307	
Age (years)	56 ± 10	48.5 ± 13.7	<0.0001
Women, *n* (%)	138 (64.2)	206 (67.1)	0.55
FPG (mg/dL)	136 (112–181)	90 (85–95)	<0.0001
HbA1c (%)	5.4 (4.4–7.5)	3.9 (3.6–4.2)	<0.0001
BMI (kg/m^2^)	30.8 ± 5	26.5 ± 4.2	<0.0001
WC (cm)	100 ± 9.9	87.4 ± 11.9	<0.0001
Men ≥ 90 cm, *n* (%)	77 (35.8)	54 (17.5)	<0.0001
Women ≥ 80 cm, *n* (%)	138 (64.2)	138 (44.9)	<0.0001
HDL-c (mg/dL)	35 (30–41)	46.4 (37.2–55.4)	<0.0001
Men < 40 mg/dL, *n* (%)	63 (29.3)	49 (15.9)	0.001
Women < 50 mg/dL, *n* (%)	126 (58.6)	111 (36.1)	<0.0001
Hypertension, *n* (%)	129 (60)	40 (13)	<0.0001
Triglycerides (mg/dL)	205 (134–270)	118 (88–150)	<0.0001
≥150 mg/dL, *n* (%)	152 (70)	79 (25.7)	
PAI-1 (ng/mL)	58.4 ± 21	49.9 ± 16	0.026
Current smoking, *n* (%)	50 (23)	62 (20.2)	0.46
FH of AT, *n* (%)	41 (19)	43 (14)	0.16

Continuous variables with normal distribution are expressed as mean ± standard deviation. Continuous variables with nonnormal distribution are expressed as median (interquartile range). Categorical variables are expressed as total number and percentages. MetS: the Metabolic Syndrome; FPG: fasting plasma glucose; HbA1c: glycosylated hemoglobin; BMI: body mass index; WC: waist circumference; HDL-c: high-density lipoprotein cholesterol; PAI-1: plasminogen activator inhibitor type-1; FH of AT: familial history of atherothrombotic disease.

**Table 2:** Genotype distribution and allele frequencies of -675 4G/5G polymorphism of the PAI-1 gene in diabetic subjects with the Metabolic Syndrome and the reference group.

	MetS	Reference group	*p*value^*∗*^
	*n* = 215	*n* = 307	
Genotype, *n* (%)			
4G/4G + 4G/5G	129 (60)	185 (60.3)	*1.0*
5G/5G	86 (40)	122 (39.7)	
4G/4G	25 (11.6)	26 (8.5)	*0.30*
4G/5G	104 (48.4)	159 (51.8)	
5G/5G	86 (40)	122 (39.7)	
Allelic frequency, *n* (%)			*0.67*
4G	154 (35.8)	211 (34.4)	
5G	276 (64.2)	403 (65.6)	

Categorical variables are expressed as total number and percentages. PAI-1: plasminogen activator inhibitor type-1; MetS: the Metabolic Syndrome; *∗*: *X*^2^ test.

**Table 3:** Correlation of PAI-1 antigen plasma concentrations with metabolic factors of diabetic subjects plus the Metabolic Syndrome.

Total = 215 subjects					
	WC	NL HDL-c	NL Tg	FPG	Hypertension
PAI-1	0.15	**−0.21**	**0.24**	0.01	**0.22**

Cells show correlation coefficients (significant in bold, *p* ≤ 0.05). PAI-1: plasminogen activator inhibitor type-1; WC: waist circumference; NL HDL-c: natural logarithm of high-density lipoprotein cholesterol; NL Tg: natural logarithm of triglycerides; FPG: fasting plasma glucose.

**Table 4:** Variability in PAI-1 antigen plasma levels explained by the components of the Metabolic Syndrome.

Explanatory variables	*β* (95% CI)	*p* value
Hypertension	0.18 (0.35 to 0.012)	*0.03*
NL triglycerides	0.15 (0.01 to 0.33)	*0.05*
NL HDL-c	−0.16 (−0.33 to −0.01)	*0.05*

*R*^2^ = 0.12, *F*-statistic = 0.001		

Explanatory variables are listed vertically; *β*: standardized correlation coefficient (95% confidence interval); *R*^2^: determination coefficient represents percentage of variance explained by the explanatory variables of the model; NL HDL-c: natural logarithm of high-density lipoprotein cholesterol; NL triglycerides: natural logarithm of triglycerides.

## References

[1] Wilson P. W. F., Kannel W. B., Silbershatz H., D'Agostino R. B. (1999). Clustering of metabolic factors and coronary heart disease. *Archives of Internal Medicine*.

[2] Kohler H. P., Grant P. J. (2000). Plasminogen-activator inhibitor type 1 and coronary artery disease. *New England Journal of Medicine*.

[3] Sobel B. E. (1999). Increased plasminogen activator inhibitor-1 and vasculopathy. A reconcilable paradox. *Circulation*.

[4] Alessi M.-C., Juhan-Vague I. (2006). PAI-1 and the metabolic syndrome: links, causes, and consequences. *Arteriosclerosis, Thrombosis, and Vascular Biology*.

[5] Klein B. E. K., Klein R., Lee K. E. (2002). Components of the metabolic syndrome and risk of cardiovascular disease and diabetes in Beaver Dam. *Diabetes Care*.

[6] Koren-Morag N., Goldbourt U., Tanne D. (2005). Relation between the metabolic syndrome and ischemic stroke or transient ischemic attack: a prospective cohort study in patients with atherosclerotic cardiovascular disease. *Stroke*.

[7] Rojas R., Aguilar-Salinas C. A., Jiménez-Corona A. (2010). Metabolic syndrome in Mexican adults: results from the National Health and Nutrition Survey 2006. *Salud Pública de México*.

[8] Isordia-Salas I., Santiago-Germán D., Rodrìguez-Navarro H. (2012). Prevalence of metabolic syndrome components in an urban mexican sample: comparison between two classifications. *Experimental Diabetes Research*.

[9] Miller A. M., Alcaraz Ruiz A., Borrayo Sánchez G., Almeida Gutiérrez E., Vargas Guzmán R. M., Jáuregui Aguilar R. (2010). Metabolic syndrome: clinical and angiographic impact on patients with acute coronary syndrome. *Cirugia y Cirujanos*.

[10] Badimon L., Hernández-Vera R., Vilahur G. (2013). Determinants of cardiovascular risk in diabetes beyond hyperglycemia. *Journal of Cardiovascular Disease*.

[11] Kullo I. J., Gau G. T., Jamil Tajik A. (2000). Novel risk factors for atherosclerosis. *Mayo Clinic Proceedings*.

[12] Isordia-Salas I., Leaños-Miranda A., Sainz I. M., Reyes-Maldonado E., Borrayo-Sánchez G. (2009). Association of the plasminogen activator inhibitor-1 gene 4G/5G polymorphism with ST elevation acute myocardial infarction in young patients. *Revista Espanola de Cardiologia*.

[13] Esparza-García J. C., Santiago-Germán D., Guadalupe Valades-Mejía M. (2015). GLU298ASP and 4G/5G polymorphisms and the risk of ischemic stroke in young individuals. *The Canadian Journal of Neurological Sciences*.

[14] Festa A., D'Agostino R., Rich S. S. (2003). Promoter (4G/5G) plasminogen activator inhibitor-1 genotype and plasminogen activator inhibitor-1 levels in blacks, Hispanics, and non-Hispanic whites: the Insulin Resistance Atherosclerosis Study. *Circulation*.

[15] Alberti K. G. M. M., Zimmet P., Shaw J. (2005). The metabolic syndrome—a new worldwide definition. *Lancet*.

[16] American Diabetes Association (2011). Diagnosis and classification of diabetes mellitus. *Diabetes Care*.

[17] Margaglione M., Grandone E., Cappucci G. (1997). An alternative method for PAI-1 promoter polymorphism (4G/5G) typing. *Thrombosis and haemostasis*.

[18] Lubrano C., Valacchi G., Specchia P. (2015). Integrated Haematological Profiles of Redox Status, Lipid, and Inflammatory Protein Biomarkers in Benign Obesity and Unhealthy Obesity with Metabolic Syndrome. *Oxidative Medicine and Cellular Longevity*.

[19] Coffey C. S., Asselbergs F. W., Hebert P. R. (2011). The association of the metabolic syndrome with PAI-1 and t-PA levels. *Cardiology Research and Practice*.

[20] Fernández-Bergés D., Consuegra-Sánchez L., Peñafiel J. (2014). Metabolic and inflammatory profiles of biomarkers in obesity, metabolic syndrome, and diabetes in a mediterranean population. DARIOS inflammatory study. *Revista Espanola de Cardiologia*.

[21] Al-Hamodi Z., Ismail I. S., Saif-Ali R., Ahmed K. A., Muniandy S. (2011). Association of plasminogen activator inhibitor-1 and tissue plasminogen activator with type 2 diabetes and metabolic syndrome in Malaysian subjects. *Cardiovascular Diabetology*.

[22] Undas A., Brummel-Ziedins K. E., Mann K. G. (2014). Anticoagulant effects of statins and their clinical implications. *Thrombosis and Haemostasis*.

[23] Cesari M., Kritchevsky S. B., Atkinson H. H. (2009). Angiotensin-converting enzyme inhibition and novel cardiovascular risk biomarkers: results from the Trial of Angiotensin Converting Enzyme Inhibition and Novel Cardiovascular Risk Factors (TRAIN) study. *American Heart Journal*.

[24] Kagami S., Kuhara T., Okada K., Kuroda Y., Border W. A., Noble N. A. (1997). Dual effects of angiotensin II on the plasminogen/plasmin system in rat mesangial cells. *Kidney International*.

[25] Saremi A., Schwenke D. C., Buchanan T. A. (2013). Pioglitazone slows progression of atherosclerosis in prediabetes independent of changes in cardiovascular risk factors. *Arteriosclerosis, Thrombosis, and Vascular Biology*.

[26] Zhang J., Noble N. A., Border W. A., Owens R. T., Huang Y. (2008). Receptor-dependent prorenin activation and induction of PAI-1 expression in vascular smooth muscle cells. *American Journal of Physiology—Endocrinology and Metabolism*.

[27] Egan B. M., Li J., Qanungo S., Wolfman T. E. (2013). Blood pressure and cholesterol control in hypertensive hypercholesterolemic patients: national health and nutrition examination surveys 1988–2010. *Circulation*.

[28] Ruiz-Quezada S., Vázquez-Del Mercado M., Parra-Rojas I. (2004). Genotype and allele frequency of PAI-1 promoter polymorphism in healthy subjects from the west of Mexico. Association with biochemical and hematological parameters. *Annales de Genetique*.

[29] De La Cruz-Mosso U., Muñoz-Valle J. F., Salgado-Bernabé A. B. (2013). Body adiposity but not insulin resistance is associated with -675 4G/5G polymorphism in the PAI-1 gene in a sample of Mexican children. *Jornal de Pediatria*.

[30] Naran N. H., Chetty N., Crowther N. J. (2008). The influence of metabolic syndrome components on plasma PAI-1 concentrations is modified by the PAI-1 4G/5G genotype and ethnicity. *Atherosclerosis*.

[31] Martínez-Calatrava M. J., Martínez-Larrad M. T., Zabena C., González-Sánchez J. L., Fernández-Pérez C., Serrano-Ríos M. (2007). The 4G/4G PAI-1 genotype is associated with elevated plasma PAI-1 levels regardless of variables of the metabolic syndrome and smoking status. A population-based study in Spanish population. *Diabetes, Obesity and Metabolism*.

[32] Kathiresan S., Gabriel S. B., Yang Q. (2005). Comprehensive survey of common genetic variation at the plasminogen activator inhibitor-1 locus and relations to circulating plasminogen activator inhibitor-1 levels. *Circulation*.

[33] Margaglione M., Cappucci G., d’Addedda M. (1998). PAI-1 plasma levels in a general population without clinical evidence of atherosclerosis: relation to environmental and genetic determinants. *Arteriosclerosis, Thrombosis, and Vascular Biology*.

[34] Morange P. E., Saut N., Alessi M. C. (2007). Association of plasminogen activator inhibitor (PAI)-1 (SERPINE1) SNPs with myocardial infarction, plasma PAI-1, and metabolic parameters: The HIFMECH Study. *Arteriosclerosis, Thrombosis, and Vascular Biology*.

[35] Isordia-Salas I., Galván-Plata M. E., Leaños-Miranda A. (2014). Proinflammatory and prothrombotic state in subjects with different glucose tolerance status before cardiovascular disease. *Journal of Diabetes Research*.

